# Computational analysis of delivery targets and trafficking at the blood‐brain barrier: toward the design of brain‐penetrant therapeutics for Alzheimer's disease

**DOI:** 10.1002/alz70859_102416

**Published:** 2025-12-25

**Authors:** Habib Baghirov

**Affiliations:** ^1^ Åbo Akademi University, Turku, Southwest Finland Finland

## Abstract

**Background:**

One approach to brain delivery relies on receptor‐mediated transcytosis (RMT). Multi‐organ atlases allow searching for RMT targets by comparing brain capillary endothelial cells (BCEC) with biodistribution‐relevant peripheral cells. I sought to create a proteomics atlas of these cells. I also analyzed protein turnover, deeming it crucial for all targets and especially those that do not transport large ligands natively and whose use in RMT may thus rely on hijacking their recycling. I then hypothesized that for targets with slow internalization, one could induce endocytosis by binding extracellular regions where conformational changes would expose possibly inactive endocytic motifs in the cytosolic tails, and sought to identify targets suitable for such triggered endocytosis and to design constructs enabling it. Finally, I reasoned that after intracellular dissociation, binding endosomal proteins may be the only way to enable faster‐than‐diffusional cargo movement, ideally abluminal, and sought to identify proteins that could intercept cargo in the endosome.

**Method:**

To create atlases, I processed the spectra of cell type‐resolved proteomics datasets and supplemented this with a transcriptomics‐based analysis. To analyze isoforms, I used peptide‐level proteomics, long‐read transcriptomics, and short‐read transcriptomics data with sufficient depth. To analyze turnover and trafficking, but also identify intracellular targets that could intercept cargo in the endosome, I used in vivo proteomics data and in vitro protein‐protein interactions and organelle fractionation datasets. To design endocytosis‐triggering constructs, I mined targets for conserved cytosolic endocytic motifs, then built allostery maps to locate suitable extracellular regions, and finally modeled binders targeting those regions.

**Result:**

Despite shallower coverage and integration challenges, the proteomics atlas allows target identification, with some deviations from transcriptomics results. Turnover data can inform target choice. Isoforms of several hits increase their specificity to BCEC or, conversely, may complicate their use. Several targets are parts of heterodimers; targeting their interface could improve specificity. The constructs designed to trigger endocytosis show high confidence scores yet require validation. Analysis of protein‐protein interactions and subcellular fractionation helps predict endosomal targets that could intercept constructs and, unlike surface proteins, would ideally be bound at acidic pH.

**Conclusion:**

These results may inform target selection and help refine constructs for better trans‐BBB delivery.

